# Cost-effectiveness of intraoperative radiation therapy versus intensity-modulated radiation therapy for the treatment of early breast cancer: a disinvestment analysis

**DOI:** 10.1186/s12913-024-10739-0

**Published:** 2024-04-03

**Authors:** Carlos Muñoz-Montecinos, Catalina González-Browne, Felipe Maza, Diego Carreño-Leiton, Pablo González, Badir Chahuan, Camila Quirland

**Affiliations:** 1 Health Technology Assessment Unit, Arturo Lopez Perez Foundation, Santiago, RM Chile; 2 Radiotherapy Department, Arturo Lopez Perez Foundation, Santiago, RM Chile; 3 Breast Surgery Unit, Arturo Lopez Perez Foundation, Santiago, RM Chile

**Keywords:** IORT, IMRT, Breast cancer, Adjuvant radiotherapy, Cost-effectiveness analysis, Disinvestment

## Abstract

**Background:**

Adjuvant radiotherapy represents a key component in curative-intent treatment for early-stage breast cancer patients. In recent years, two accelerated partial breast irradiation (APBI) techniques are preferred for this population in our organization: electron-based Intraoperative radiation therapy (IORT) and Linac-based External Beam Radiotherapy, particularly Intensity-modulated radiation therapy (IMRT). Recently published long-term follow-up data evaluating these technologies have motivated a health technology reassessment of IORT compared to IMRT.

**Methods:**

We developed a Markov model to simulate health-state transitions from a cohort of women with early-stage breast cancer, after lumpectomy and adjuvant APBI using either IORT or IMRT techniques. The cost-effectiveness from a private health provider perspective was assessed from a disinvestment point of view, using life-years (LYs) and recurrence-free life-years (RFLYs) as measure of benefits, along with their respective quality adjustments. Expected costs and benefits, and the incremental cost-effectiveness ratio (ICER) were reported. Finally, a sensitivity and scenario analyses were performed to evaluate the cost-effectiveness using lower IORT local recurrence and metastasis rates in IORT patients, and if equipment maintenance costs are removed.

**Results:**

IORT technology was dominated by IMRT in all cases (i.e., fewer benefits with greater costs). Despite small differences were found regarding benefits, especially for LYs, costs were considerably higher for IORT. For sensitivity analyses with lower recurrence and metastasis rates for IORT, and scenario analyses without equipment maintenance costs, IORT was still dominated by IMRT.

**Conclusions:**

For this cohort of patients, IMRT was, at least, non-inferior to IORT in terms of expected benefits, with considerably lower costs. As a result, IORT disinvestment should be considered, favoring the use of IMRT in these patients.

**Supplementary Information:**

The online version contains supplementary material available at 10.1186/s12913-024-10739-0.

## Background

Early-stage breast cancer paradigm treatment consists of lumpectomy followed by adjuvant whole breast irradiation (WBI) [1]. This is a successful and well-tolerated treatment that has demonstrated non-inferiority to radical mastectomy, with an improvement in women’s quality of life [2–4]. Despite these benefits, a decade before, standard WBI guidelines suggested 5 weeks + 1 or 2 additional weeks for boost [5], which represents costs and logistical issues that many patients are unable to deal with, thus forgoing therapy [6].

Accelerated partial breast irradiation (APBI) has been developed as an alternative treatment method to deliver localized radiotherapy to the area at the highest risk of recurrence in a shorter time compared to WBI. In APBI, irradiation is delivered to the tumor surrounding tissue, reducing radiation toxicity to adjacent organs such as the heart and lungs. One technique for APBI is Intraoperative radiation therapy (IORT), which refers to the delivery of a single dose of radiation directly to the tumor bed at the time of surgery. In addition to the benefits of other APBI techniques, IORT eliminates the risk of patients not attending to prescribed adjuvant radiotherapy due to logistic issues.

IORT efficacy and safety have been evaluated in Electron Intraoperative Radiotherapy (ELIOT) and TARGIT-A randomized controlled trials [7, 8]. After 10 years of being followed, patients on the IORT arm showed no inferiority in terms of overall survival compared to WBI. However, an increase in local recurrence was observed in IORT patients, which has led some authors to question the benefits of IORT over not using radiotherapy at all [9, 10].

Another APBI method is the intensity-modulated radiation therapy (IMRT), which is based on the delivery of nonuniform fluence and can deliver a highly conformed dose to the target. For early breast cancer patients, long-term results from the FLORENCE trial showed no differences between IMRT delivered at 5 once-daily fractions and WBI in 10-year ipsilateral breast tumor recurrence and overall survival, with improvements in toxicity and cosmesis-related outcomes [11].

In our organization, equipment able to deliver electron-based IORT (LIAC, Sordina) arrived in 2012 when no other radiotherapy option besides WBI was available. It meant an important treatment alternative for women unable to attend WBI schedules. However, a few years after, equipment with the ability to deliver IMRT (TomoTherapy Hi-Art, Accuracy and Synergy VMAT, Elekta) was acquired, increasing treatment options for early breast cancer patients. Since the implementation of APBI with IMRT in our organization, clinicians have favored its use over IORT, mainly because of its lower recurrence rate and the possibility to delivering radiotherapy within one week. Considering the long-term results from IORT trials, the low number of patients currently treated with IORT and the high maintenance costs of the equipment, the Health Technology Assessment Unit of our organization performed a reevaluation of the expected benefits and costs of IORT compared with IMRT. To this end, a disinvestment analysis was performed to determine the best use of our resources, by either keeping IORT available, bearing the maintenance expenses, or shifting the expenditure towards IMRT, utterly displacing IORT as a treatment option. Using data from ELIOT and FLORENCE trials [7, 11], a Markov model was developed to estimate the cost-effectiveness of IORT in patients with early-stage breast cancer in comparison with IMRT.

## Methods

A cost-effectiveness analysis was developed through a Markov model to compare the expected benefits and costs of patients undergoing IORT or IMRT after breast conservative surgery. Given that our organization is an oncological institute, our analysis was conducted from the perspective of a private health provider as a disinvestment decision analysis. Benefits are reported using four different outcomes: life years (LYs), quality-adjusted life years (QALYs), recurrence-free life years (RFLYs), and quality-adjusted recurrence-free life years (QARFLYs). Results of the cost-effectiveness analysis are reported using the incremental cost-effectiveness ratio (ICER).

### Decision model

**Fig. 1:**
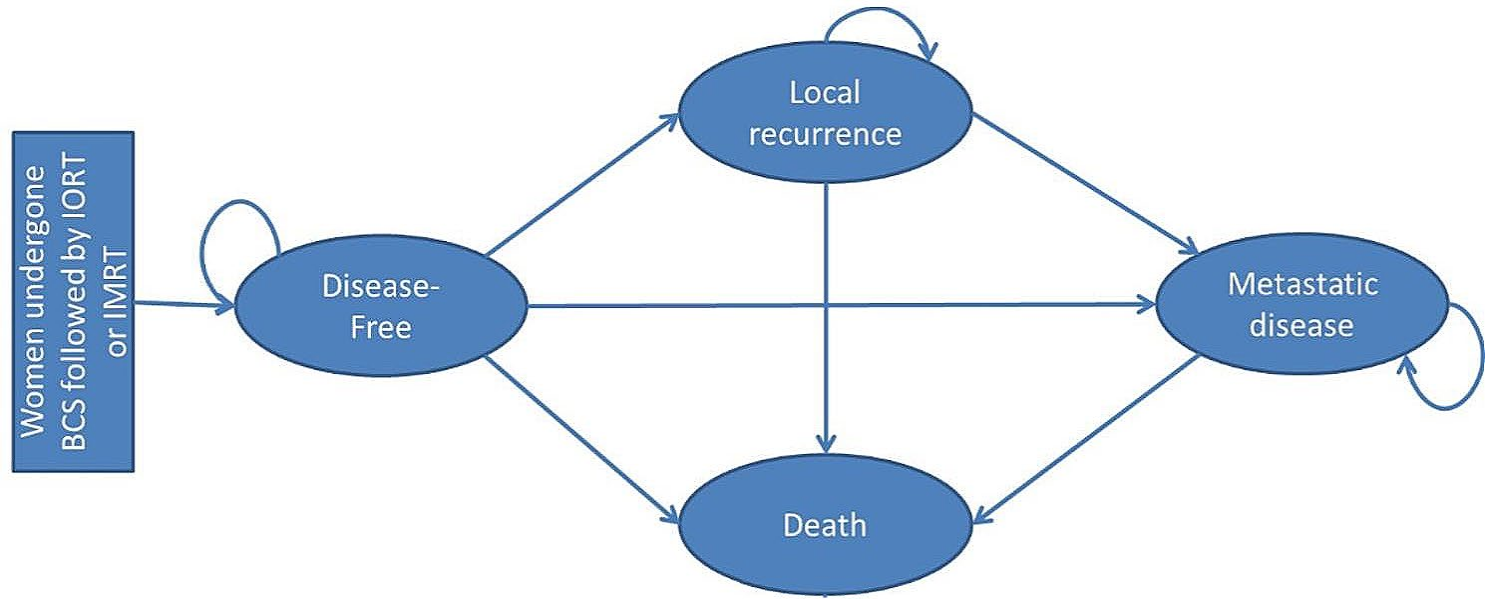
Schematic representation of the Markov model. Each oval represents a health state. At the beginning, all women are in a disease-free state, being able to remain there or transition to other health states as indicated by arrows. BCS = breast conservative surgery; IORT = intraoperative radiation therapy; IMRT = intensity-modulated radiation therapy

The Markov model was designed to simulate the clinical history of women diagnosed with early breast cancer after undergoing breast conservative surgery and either IORT or IMRT as an adjuvant radiotherapy treatment using standard doses. The model is composed of 4 health states: disease-free, local recurrence, metastatic disease, and death. A cohort of 100 women aged 60 years old was used to go through the health states of the model in annual cycles. All patients started at the disease-free health state, and they may remain disease-free or may transition to one of the other mutually exclusive health states: local recurrence, metastasis or death, the latter related or not to breast cancer (Fig. 1).

The simulation was performed through a time horizon of 10 years, and we applied a discount rate of 3% for benefits and costs, as it is recommended in the Methodological guidelines for the economic evaluation of health intervention in Chile [12]. The model was developed in Microsoft Excel (version 16.63.1).

### Model data input

Despite the fact that IORT was implemented in our organization in 2012, we decided not to use our patient’s data to inform the model because, at that time, IORT was used mainly for boost after WBI, and later it was used for exclusive APBI. Additionally, the patient eligibility criteria have changed over time, where the current ASTRO/GEC-ESTRO recommendations were used in just a few patients and the follow-up time is not long enough to obtain mature data to inform the model [13, 14]. For this reason, IORT and IMRT transition probabilities for local recurrence, metastasis, and death due to breast cancer were obtained from the trials ELIOT and FLORENCE [7, 11], respectively, considering that their radiotherapy schemes are similar to those used at our organization. Probabilities of death due to any cause for women aged 60 or more, in 5-year intervals, were obtained from the National Statistics Institute of Chile [15].

Transitions to local recurrence and mortality due to breast cancer were included in the model as time-dependent probabilities, using ipsilateral breast tumor recurrence (IBTR) and overall survival curves, respectively, both reported in ELIOT and FLORENCE trials [7, 11]. Annual probabilities were estimated from cumulative probabilities of each survival curve. Metastasis risk from the disease-free state was included as a fixed probability using 10-year rates reported in the trials [7, 11], since no survival curve was reported in the ELIOT trial for this outcome. In addition, it was assumed a 3% annual rate of metastasis from local recurrence, based on results observed in a study with a similar cohort of women [16].

Mean utility values for each health state were obtained from a published study on patient preferences [17]. All transition probabilities are listed in Table 1.

**Table 1:** Model parameters for Intraoperative radiation therapy (IORT) and Intensity-modulated radiation therapy (IMRT)

	Base case		Sensitivity analysis	Source
	IORT	IMRT	IORT	
**Transition probabilities**				
LR after BCS year 1	0.000	0.002	0.000	[7, 11]
LR after BCS year 2	0.001	0.004	0.000	[7, 11]
LR after BCS year 3	0.018	0.004	0.010	[7, 11]
LR after BCS year 4	0.009	0.011	0.008	[7, 11]
LR after BCS year 5	0.005	0.000	0.006	[7, 11]
LR after BCS year 6	0.016	0.000	0.011	[7, 11]
LR after BCS year 7	0.001	0.004	0.002	[7, 11]
LR after BCS year 8	0.012	0.008	0.010	[7, 11]
LR after BCS year 9	0.010	0.000	0.008	[7, 11]
LR after BCS year 10	0.006	0.000	0.005	[7, 11]
Metastasis after LR	0.03	0.03	–	[16]
Metastasis after RF	0.006	0.0029	0.004	[7, 11]
Death by any cause 50–54	0.002			[15]
Death by any cause 55–59	0.004			[15]
Death by any cause 60–64	0.006			[15]
Death by any cause 65–69	0.009			[15]
Death by breast cancer year 1	0.003	0.004		[7, 11]
Death by breast cancer year 2	0.002	0.004		[7, 11]
Death by breast cancer year 3	0.006	0.006		[7, 11]
Death by breast cancer year 4	0.008	0.006		[7, 11]
Death by breast cancer year 5	0.017	0.006		[7, 11]
Death by breast cancer year 6	0.018	0.003		[7, 11]
Death by breast cancer year 7	0.006	0.012		[7, 11]
Death by breast cancer year 8	0.011	0.010		[7, 11]
Death by breast cancer year 9	0.008	0.017		[7, 11]
Death by breast cancer year 10	0.018	0.005		[7, 11]
**Costs**				
Treatment cost	$418	$29		–
Other direct cost	$359	$294		–
Annual maintenance cost	$55,344	–		–
**Health state utilities**				
Disease free	0.920			[17]
Local recurrence	0.779			[17]
Other recurrences	0.685			[17]

*Abbreviations* BCS = Breast conservative surgery; LR = Local recurrence; RF = Recurrence free

### Cost data

Only direct costs were considered for the analysis. These costs were represented by treatment costs and other direct costs. Treatment costs include equipment and operating room usage, the latter only for IORT. On the other hand, other direct costs include pretreatment and treatment supervision staff fees (medical physicist, medical technologist, and radiation therapist). Only in the case of IMRT, the costs of medical and nursing appointments between radiation fractions were added, along with a final consult for discharge from treatment. Follow-up costs were not included for IORT, as patients have a follow-up with the breast surgeon only. Additionally, maintenance costs of IMRT were not included since the same equipment is used for other indications related to other health conditions, and the reallocation of IORT patients to be treated with IMRT does not increase the costs of its maintenance. Moreover, the IMRT equipment has had sufficient capacity to receive the small number of IORT patients treated annually (10–12 patients).

Since our analysis is focused on a disinvestment decision related to one of two pieces of equipment that are already paid in the organization, the costs included are associated only with its use, excluding the initial investment. Costs related to complications were not included in the analysis, as they were assumed to be equal for both interventions. Treatment costs, other direct costs per patient and maintenance costs are listed in Table 1. Unit costs are from our organization and are expressed in 2021 USD (1 USD = 786 CLP).

### Sensitivity and scenario analysis

Two one-way deterministic sensitivity analyses were conducted in order to evaluate the consistency of the results. IORT model-input parameters for local recurrence and metastasis rates were varied to the lower limit of the confidence interval of the curve and 10-year rate, respectively, reported in ELIOT trial [7].

One additional scenario was simulated, where only the costs were modified. In this scenario, maintenance costs of IORT were excluded from the analysis to evaluate how important they were in the results obtained, since maintenance costs of IMRT were excluded in the base case.

## Results

### Model validation

We performed an external validation of our model results by comparing them against those previously published or predicted by web tools. Our model predicted a 10-year local recurrence rate of 7.17%, while another cost-effectiveness analysis with a similar cohort of patients reported 10-year local-recurrence rates of 7.45% [18]. Regarding the OS, we estimated a 92.57% 10-year survival rate, which is comparable to 86.5% and 85.13% obtained in previous cost-effectiveness studies [18, 19]. Additionally, we compared our predicted OS with a 10-year OS predicted by Predict, a population-based online tool to predict survival for early-stage breast cancer patients (https://breast.predict.nhs.uk/tool) [20]. This tool estimates a 10-year OS of 89% and 85% for tumor sizes of 10 and 20 mm, respectively.

### Base case

In our base cost-effectiveness analysis, the IORT strategy is dominated by IMRT for all outcomes. Given that there are almost no differences in OS between both radiotherapy techniques, our model delivers a very small difference in LYs in favor of IMRT, resulting in a negative ICER value given the higher costs of IORT (Table 2). When using RFLYs, and in accordance with the local recurrence data reported, a marginally greater benefit is observed for IMRT compared to IORT, resulting in an incremental benefit of −0.27. These results are consistent irrespective of the adjustment for quality of LYs and RFLYs, obtaining an incremental value of −0.05 and − 0.25 for QALYs and QARFLYs, respectively. Regarding costs, IORT results in an incremental expected cost of $USD 1,006 in relation to IMRT.

Taken together the results of these outcomes and the expected costs, ICERs are negative, showing the non-inferiority of IMRT in terms of clinical benefits, accompanied by a lower cost per patient (Table 2).

**Table 2:** Results of the base case, sensitivity and scenario analysis for the comparison between Intraoperative radiation therapy (IORT) and Intensity-modulated radiation therapy (IMRT). Expected benefits and costs, together with the corresponding ICER, are reported for each case and outcome

	IORT	IMRT	Incremental	ICER
**Base case**				
LYs	8.48	8.48	−0.002	−$426,358
RFLYs	7.92	8.19	−0.27	−$3,733
QALYs	7.69	7.75	−0.05	−$19,095
QARFLYs	7.29	7.53	−0.25	−$4,058
Costs	$1,330	$323	$1,006	–
**Sensitivity analysis**				
*IORT lower recurrence*				
LYs	8.48	8.48	−0.002	−$432,972
RFLYs	8.01	8.19	−0.18	−$5,531
QALYs	7.71	7.75	−0.04	−$25,309
QARFLYs	7.37	7.53	−0.17	−$6,012
Costs	$1,330	$323	$1,006	–
*IORT lower metastasis*				
LYs	8.48	8.48	−0.002	−$566,948
RFLYs	7.99	8.19	−0.20	−$4,939
QALYs	7.71	7.75	−0.04	−$27,438
QARFLYs	7.35	7.53	−0.19	−$5,369
Costs	$1,330	$323	$1,006	–
**Scenario analysis**				
LYs	8.48	8.48	−0.002	−$191,905
RFLYs	7.92	8.19	−0.27	−$1,680
QALYs	7.69	7.75	−0.05	−$8,595
QARFLYs	7.29	7.53	−0.25	−$1,826
Costs	$776	$323	$453	–

*Abbreviations* LYs = Life years, RFLYs = Recurrence-free life years, QALYs = quality-adjusted life years, QARFLYs = Quality-adjusted recurrence-free life years

### Sensitivity and scenario analysis

**Fig. 2:**
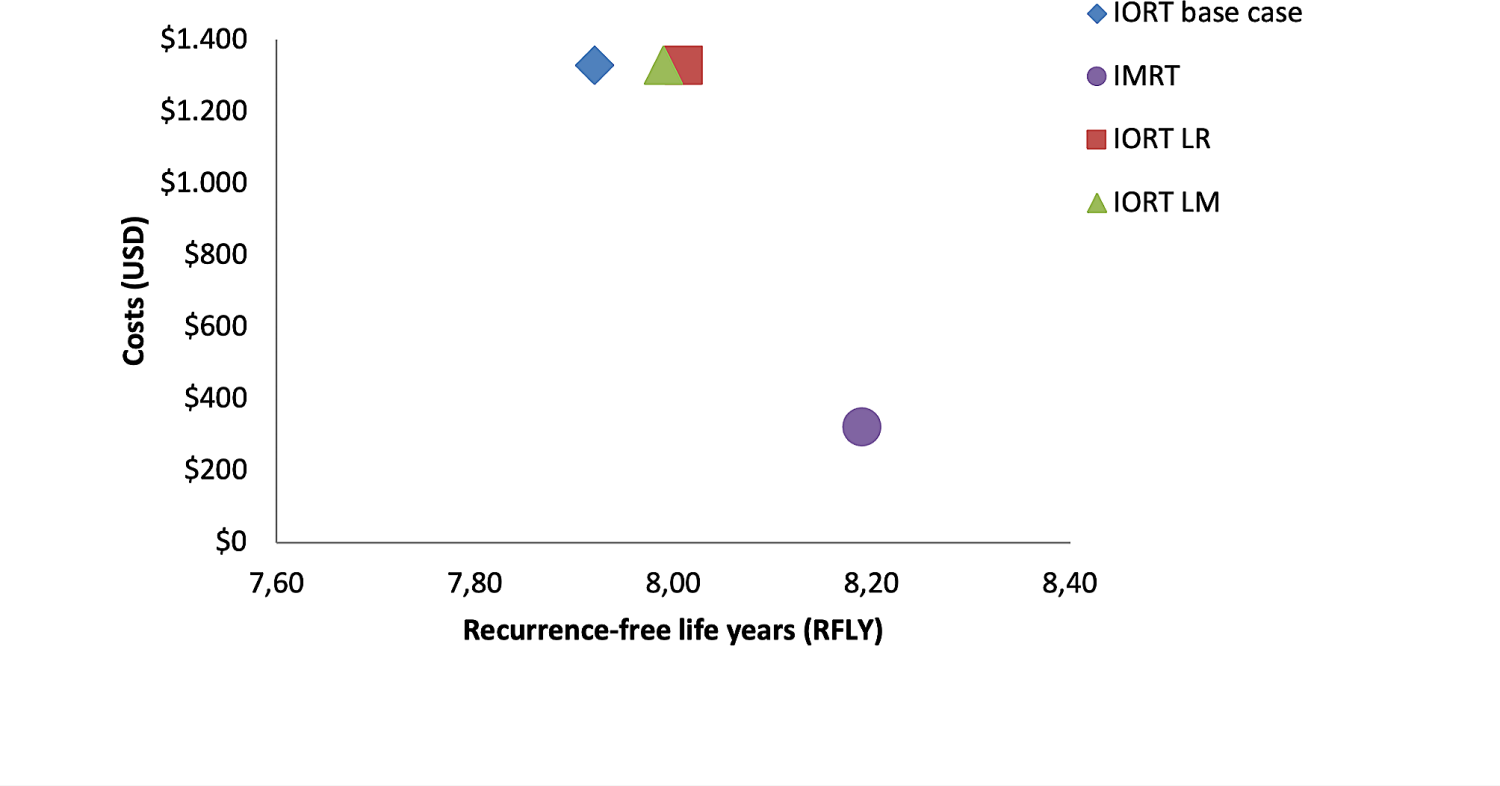
Cost-effectiveness graph of IORT in base case and sensitivity analysis compared to IMRT. Regardless of IORT scenario, IMRT has the greatest benefits at the lowest costs. Abbreviations: IMRT= intensity-modulated radiation therapy; IORT= intraoperative radiation therapy; LR= lower local recurrence rate; LM= lower metastasis rate

A sensitivity analysis, in which IORT local recurrence and metastasis rate were adjusted to the lower limit of confidence interval reported in the ELIOT trial, was performed. Although both rates were decreased, in both cases IORT technology was dominated by IMRT, reinforcing the conclusions obtained in the base case (Fig. 2; Table 2).

The results of the alternative scenario in which IORT maintenance costs were excluded are also shown in Table 2. The expected benefits are the same as in the base case, as only costs were varied. Resulting ICERs lead to indistinguishable conclusions to the base case, although one additional unit of benefit results in a lower incremental cost when maintenance costs of IORT are excluded.

## Discussion

Our study is, to our knowledge, the first cost-effectiveness analysis comparing IORT against IMRT as adjuvant treatment for women with early-stage breast cancer who underwent breast-conservative surgery, from a disinvestment point of view. Although IORT is used in other cancer types, we focused our analysis on this population as it represents the largest proportion of patients treated with this technology in our organization. Moreover, the best quality of evidence available for IORT is in this group of patients.

Health Technology Reassessment (HTR) is an emerging field involving the assessment of a technology currently in use in a healthcare system, to inform its optimal use compared with the alternatives [21]. This reassessment was done from a disinvestment approach to reallocating the resources destined to cover IORT maintenance expenses to better treatment options, like IMRT. This decision would not only result in a disinvestment decision but a de-adoption as well, given the elimination of the IORT system would make the practice of IORT unavailable altogether [22].

We decided to perform this study due to some concerns that recently arose regarding IORT technology: the results of the long-term follow-up from TARGIT-A and ELIOT trials [7, 8], and the low number of patients undergoing IORT in our organization after the acquisition of IMRT technology.

The long-term results of the TARGIT-A and ELIOT trials [7, 8] were recently published, showing a higher rate of local recurrence in women treated with IORT compared to WBI. In the ELIOT trial, the 10-year ipsilateral breast tumor recurrence (IBTR) rates in the IORT arm and WBI arm were 8.1% (95% CI 6.1–10.3) and 1.1% (0.5–2.2) respectively, being the difference statistically significant [7]. Similar results emerged from the TARGIT-A trial, although the authors did not report a 10-year local recurrence rate in publication of the long-term results, and only reported an updated 5-year local recurrence rate, being 2.11% for the IORT arm and 0.95% for WBI (difference 1.16%, 90% CI 0.32 to 1.99) [8]. Despite this being a small difference, some elements should be considered: in the TARGIT-A protocol, some patients with high-risk of recurrence received WBI in addition to IORT. Furthermore, even though the *p* value for local recurrence was not reported, Sasieni and Sawyer calculated it, obtaining a significant difference in local recurrence between IORT and WBI. The same authors, and others, argue that, in terms of local recurrence, IORT is inferior to WBI, and probably it is no better than no radiotherapy at all [10].

On the other hand, long-term results from the FLORENCE trial showed no statistically significant difference in 10-year cumulative incidence in IBTR for the APBI arm compared to WBI (3.7% vs. 2.5%, *p* = 0.4) [11]. Regarding the long-term results in overall survival, neither IORT nor IMRT studies showed significant differences compared to WBI.

The ICER values obtained in our model showed that IORT is dominated by IMRT in all cases. Considering the small difference in benefits mentioned, the ICER values are mainly explained by high differences in costs associated with each technology. Those costs are higher for IORT in all dimensions shown in Table 1. This is evidenced by the scenario analysis, where the elimination of maintenance costs, which represent the highest individual cost, preserves the cost differences in favor of IMRT, maintaining the meaning of the result unchanged. Moreover, when some parameters related to benefits were varied in sensitivity analysis, the results continue to be consistent with the base case, which confirms the robustness of our results.

Several cost-effectiveness analyses regarding the use of IORT or IMRT in breast cancer patients have been performed in the last years, but none compares both technologies from a disinvestment point of view. For the IORT technology, a systematic review of articles comparing IORT with external beam radiation therapy (EBRT) was done by Eisavi et al., who found 8 cost-effectiveness articles that met the selection criteria. Inconsistent results regarding IORT cost-effectiveness were reported: 4 articles show that IORT costs were lower with more benefits compared to EBRT, while in 3, despite IORT costs being also lower than EBRT, the benefits were lower too and it was dominated by EBRT [23]. Some of the studies that reported more benefits for IORT extrapolated local recurrence rate at years from 5 years data reported in the TARGIT-A trial, which lead to estimation errors. For example, Alvarado et al. projected a 10-year local recurrence rate of 2.4% for whole-breast EBRT arm, more than 2 times the local recurrence rate reported in the long-term follow-up of the ELIOT trial [19]. Patel et al. also reported that IORT dominates EBRT, but they projected a cumulative probability of events for recurrent cancer and death for IORT of 23% vs. EBRT 25.4% [24], which is not consistent with the recently published follow-ups of ELIOT and TARGIT-A trials. On the other hand, we found very few articles about IMRT cost-effectiveness, and, to our knowledge, none of them evaluated IMRT in a clinical context similar to our study.

Only one article compared IORT against IMRT and other radiotherapy technologies. In that study, which incorporated medical and nonmedical costs (in USD), the ICER calculated for IORT vs. IMRT ranged $244–$433 and $788–$1178 based on local recurrence rates from ELIOT and TARGIT-A respectively, which means that IMRT is a cost-effective technique compared to IORT if a willingness-to-pay threshold per percentage point of reduction in local recurrence of $1,000–$2,000 is used [25]. IMRT was also cost-effective compared to IORT based on cost-per-QALY analyses, obtaining costs of $17,335/QALY–$29,347/QALY, which are below the commonly used thresholds to define cost-efficacy in breast cancer. It is worth mentioning that in this study, IORT costs are lower than IMRT costs, contrary to our values. It occurs because in their article costs were not estimated from a private health provider perspective.

One of the main limitations of our study is related to the source of information of the parameters used to inform the model. Our analysis was approached as a disinvestment evaluation of a technology already acquired and implemented in the institution. Despite this fact, we have not been able to use our patients’ data to estimate the outcomes required to feed the model. Changes in modality, doses and target population of IORT for early-stage breast cancer treatment in clinical guidelines recommendations [13, 26, 27] have made it impossible to gather a large enough cohort of patients with an adequate follow-up to estimate the parameter using local data. For this reason, we decided to use the outcomes reported in the ELIOT and FLORENCE trials, the ones that best reflect the local clinical practice. Additional to this, utility values were also obtained from published evidence and not estimated locally. The source of information was a study conducted by Hayman et al. in 1997 [17] where patient preferences related to radiotherapy after breast conservative surgery were collected. Although the target population is the same as that of the present evaluation, preferences are jurisdiction-dependent and should be estimated locally. This limitation only affects quality-adjusted outcomes, where more studies revealing local preferences are needed to have more precise estimates of them.

Another limitation of our analysis is that we did not consider radiotherapy effects beyond disease control. This means that no costs related to complications or adverse events related to radiotherapy were included as they were assumed to be equal for both interventions.

## Conclusions

In summary, the disinvestment of electron-based IORT equipment should be considered by decision-makers, given its higher costs and non-superiority regarding benefits compared to IMRT for the treatment of early-stage breast cancer patients.

### Electronic supplementary material

Below is the link to the electronic supplementary material.

Supplementary Material 1

## Data Availability

Data generated or analysed during this study are included in this published article.

## Abbreviations

APBI: Accelerated partial breast irradiation
BCS: Breast conservative surgery
EBRT: External beam radiation therapy
ELIOT: Electron intraoperative radiotherapy
HTR: Health Technology Reassessment
IBTR: Ipsilateral breast tumor recurrence
ICER: Incremental cost-effectiveness ratio
IMRT: Intensity-modulated radiation therapy
IORT: Intraoperative radiation therapy
LM: Lower metastasis rate
LR: Lower local recurrence rate
LYs: Life-years
QALYs: Quality-adjusted life years
QARFLYs: Quality-adjusted recurrence-free life years
RFLYs: Recurrence-free life-years
WBI: Whole breast irradiation
